# *In vivo* evaluation of DSAEK interface with scanning-laser confocal microscopy

**DOI:** 10.1186/1471-2415-12-32

**Published:** 2012-08-01

**Authors:** Giulio Ferrari, Verena Reichegger, Luca Ludergnani, Elisabetta Delfini, Claudio Macaluso

**Affiliations:** 1 G.B. Bietti Eye Foundation, IRCCS, Rome, Italy; 2 Dept. of Ophthalmology, University Hospital of Parma, Parma, Italy

**Keywords:** DSAEK, Interface, Laser corneal confocal microscopy

## Abstract

**Background:**

Descemet Stripping Automated Endothelial Keratoplasty (DSAEK) allows selective replacement of the endothelium. Post-operative haze and particles can affect the interface quality and, ultimately, visual outcome. In this study, we evaluated DSAEK interface with *in vivo* laser confocal microscopy (LCM) in order to: (i) correlate interface status with best corrected visual acuity, and (ii) with time from surgery; (iii) correlate interface particle number with best corrected visual acuity. Host-donor interface was imaged and graded using a published reflectivity scale. Particles at the interface were counted.

**Methods:**

18 eyes of 16 patients (6 males and 10 females); mean age: 74 ± 8.3 years which underwent DSAEK were examined by means of *in vivo* laser confocal microscopy between 1 and 24 months after surgery. Host-donor interface was imaged and graded using a published reflectivity scale. Particles present at the interface were counted.

**Results:**

Interface reflectivity was 2.17 ± 1.2 and significantly correlated with visual acuity (Spearman correlation coefficient −0.83; P < 0.001), and with time after surgery (Spearman correlation coefficient −0.87; P < 0.001). Visual acuity was 0.67 ± 0.27. The number of particles was 205 ± 117.8; no correlation was found between this number and visual acuity (Spearman correlation coefficient −0.41; P = 0.15).

**Conclusion:**

DSAEK interface imaged with LCM is helpful in diagnosing poor host-donor interface quality in DSAEK surgery. A good quality interface is related to a better visual acuity. Moreover, the quality of the interface appears to improve as time passes from the surgery. Interface quality is related with visual acuity and improves with time from surgery. LCM should be considered as an added tool in post-DSAEK follow-up of patients. Finally, our study shows that the presence of particles does not influence visual outcome.

## Background

Descemet Stripping Automated Endothelial Keratoplasty (DSAEK) is a recently developed lamellar corneal transplant which allows selective replacement of the endothelial side of the cornea. It has been proposed as an alternative to penetrating keratoplasty (PK) in cases of dysfunctional endothelium [1-3]. DSAEK has gained increasing popularity as it induces less post-operative astigmatism, less high order aberrations, and encounters higher patient satisfaction, when compared to PK [4]. Differently from PK, however, DSAEK creates a donor-host interface which can cause significant haze and possibly influence visual recovery. Moreover, particles of different nature (lint, cellular debris, plastic or metallic particles) can be detected often times at the slit-lamp and their possible impact on visual acuity has been object of concern [5].

A new generation of *in-vivo* laser confocal microscopy devices (Heidelberg Retina Tomograph 2 Rostock Cornea Module; Heidelberg Engineering GmbH, Dossenheim, Germany) has become available recently [6-8]. These instruments provide histology-quality images, without the need to remove, stain and cut the tissue, with a higher axial resolution (4 μm) than that achieved with white-light confocal microscopy (10 μm with ConfoScan 2, Nidek Technologies, Vigonza, Italy). This case series investigates the impact of interface reflectivity and particles on visual acuity in 16 patients at different time-points. Patients were consecutively enrolled during a two-month time frame.

## Methods

The ophthalmology department board approved this study. Informed consent was obtained prior to performing confocal microscopy; the study followed the tenets of the Declaration of Helsinki. 18 eyes of 16 patients (6 males and 10 females) who underwent DSAEK surgery due to Fuchs’ dystrophy at the University Hospital of Parma, Italy were included in the study. Patients were proposed to join the study and the protocol was explained.

Exclusion criteria for this study were glaucoma, ocular infections and uveitis. Patients were imaged between 1 and 24 months following DSAEK surgery.

All patients underwent a complete ophthalmology visit including refraction with determination of ETDRS-measured Best Spectacle Corrected Visual Acuity (BSCVA), biomicroscopy, intraocular pressure measurement, and in-vivo scanning laser confocal microscopy (LCM).

Patients underwent DSAEK surgery alone or associated with cataract extraction (Table 1).

**Table 1:** Demographics of the study population

**Patient**	**Age**	**Gender**	**Gender Diagnosis**	**Procedure**	**BSCVA (Snellen)**
1	83	M	Fuchs′	DSAEK	1
2	56	M	Fuchs′	DSAEK	0.8
3	78	F	Fuchs′ + cataract	DSAEK + FACO + IOL	0.6
4	72	F	Fuchs′	DSAEK	0.8
5*	65	M	Fuchs′ + cataract	DSAEK + FACO + IOL	1
6*	65	M	Fuchs′	DSAEK	0.8
7*	72	F	Fuchs′	DSAEK	0.8
8*	72	F	Fuchs′ + cataract	DSAEK + FACO + IOL	0.8
9	77	M	Fuchs′	DSAEK	1
10	85	F	Fuchs′	DSAEK	0.6
11	69	F	Fuchs′	DSAEK	0.8
12	82	F	Fuchs′	DSAEK	0.4
13	78	F	Fuchs′	DSAEK	0.13
14	77	F	Fuchs′	DSAEK	0.4
15	83	F	Fuchs′	DSAEK	0.2
16	68	F	Fuchs′	DSAEK	1
17	85	M	Fuchs′	DSAEK	0.4
18	65	M	Fuchs′	DSAEK	0.6

Asterisks indicate eyes from the same patient (5, 6 and 7, 8).

### DSAEK surgery

All procedures were performed under monitored anesthesia with peribulbar block, following a published technique [9]. Briefly, donor lenticules were prepared with a Moria artificial chamber, a Carriazo-Barraquer microkeratome (300 μm microkeratome head), and a punching block (Moria, Antony, France). The anterior chamber of the recipient eye was then entered through a 4 to 5 mm clear corneal incision. An anterior chamber maintainer was used to prevent anterior chamber collapse during surgery. Descemet membrane was stripped from the central 8 mm. The peripheral edge of the rolled endothelial graft was grasped from a device inserted in the corneal tunnel and pulled inside the anterior chamber with either coaxial forceps or a Prolene 10–0 suture. A small air bubble was injected to lift the donor tissue. After centering the graft, the anterior chamber was completely filled with air. After 10 minutes, the air bubble was reduced to about 80% of the size of the endothelial graft.

### *In vivo* confocal microscopy

All eyes were examined using the same LCM device (Heidelberg Retina Tomograph 2 Rostock Cornea Module; Heidelberg Engineering GmbH, Dossenheim, Germany). Briefly, patients were instilled with a drop of topical anesthetic (Oxybuprocaine chloridrate 0.4%) 5 minutes and immediately before the exam. The patient was then asked to steadily fixate a target and LCM was performed on the central cornea as we described previously [10]. The exam lasted approximately 10 minutes. At the end of the examination, antibiotic ointment (Ofloxacin 0.3%) was applied.

Examination was performed taking scans of the epithelium, and progressively focusing to deeper layers down to the interface area. Interface area was identified as the acellular zone found proceeding from the epithelium to the endothelium. This area hosted often times birefringent particles, as previously described (11). At least three scans - chosen for image quality - were considered. Images obtained were 400x400 microns wide. Particle density was measured using a built-in software (Cornea section cell count; Heidelberg Engineering), which calculates the density of objects manually selected by the operator. In order to minimize variability, a single investigator blinded to the patient group analyzed and graded all the images retrospectively. Images were graded following a published method specifically studied for HRT2 confocal microscope [11]. The grading was performed by comparing our images with sample images provided by Kobayashi et al. We used a 4 grade scale (grade 1: no haze; grade 4: severe haze).

### Statistical analysis

Data analysis was performed using SPSS for Windows (version 11.0, SPSS Inc, Chicago, Illinois). Correlation was studied by means of Spearman Correlation Coefficient. Difference was considered statistically significant if the P value was <0.05, and highly significant if P was <0.01.

## Results and discussion

We examined 6 males and 10 females (16 patients, 18 eyes). The mean age was 74 ± 8.3 and ranged from 56 to 85 years. Table 1 provides the demographics of the study population.

**Figure 1:**
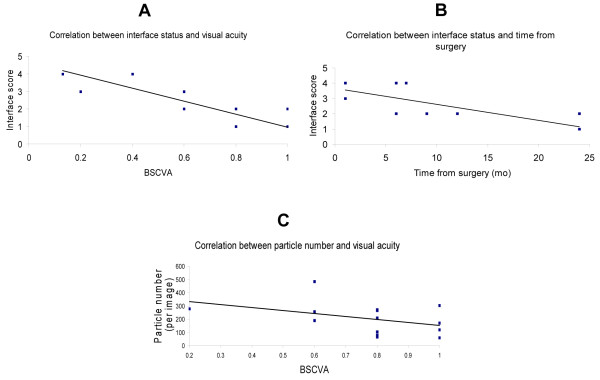
**A. Negative correlation between interface reflectivity and BSCVA.** The higher the interface reflectivity, the lower the visual acuity (Spearman correlation coefficient −0.83; P < 0.001) **B.** Negative correlation between interface reflectivity and with time after surgery (Spearman correlation coefficient −0.87; P < 0.001). The longer the time from surgery, the lower the interface reflectivity. **C.** No correlation was found between the number of particles at the interface and visual acuity (Spearman correlation coefficient −0.41; P = 0.15).

Interface reflectivity was 2.17 ± 1.2 and significantly correlated with visual acuity (Spearman correlation coefficient −0.83; P < 0.001), and with time after surgery (Spearman correlation coefficient −0.87; P < 0.001); Figure 1 A and B.

Visual acuity averaged over all the patients was 0.67 ± 0.27. The number of particles was 205 ± 117.8. No correlation was found between the number of particles at the interface and visual acuity (Spearman correlation coefficient −0.41; P = 0.15); Figure 1C.

Moreover, we did not find a significant correlation between the time from surgery and the number of interface particles (Spearman coefficient 0.53, P = 0.06).

**Figure 2:**
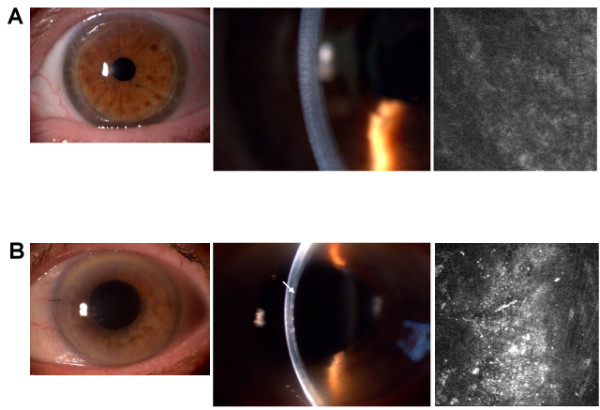
**Panel A. Representative slit lamp and confocal pictures of a low reflectivity (i.e. good quality) interface.** Note the clear interface observed at the slit lamp, which corresponds to a dark confocal image. The BSCVA in this patient was 20/20. **Panel B.** Representative slit lamp and confocal pictures of a high reflectivity (i.e. poor quality) interface. Note the evident white line representing the donor-host interface (arrow), which corresponds to a hazy confocal picture. The BSCVA in this patient was 20/40.

Interface quality assessment with slit-lamp and LCM appeared consistent (Figure 2).

DSAEK surgery is considered a valuable alternative to PK in cases of dysfunctional endothelium such as in Fuchs’ dystrophy as it selectively replaces it. DSAEK is less invasive and better tolerated by patients [4], allows prompt improvement and stability of visual acuity. However, being a lamellar surgery, it creates an interface between the donor and the recipient tissue, where the healing process is thought to be critical to optimal visual recovery [11]. DSAEK grafts present increased haze at least up to three months, when compared to PK grafts [12]. Loss of corneal transparency and swelling in Fuch’s dystrophy have been object of extensive studies [13].

The most common cause of DSAEK failures is secondary to endothelial cell loss, followed by “dysfunctional” donor-host interface. These include lenticule dislocation, interface fibrosis or hemorrhage, epithelial downgrowth [14-16].

LCM is a low-invasivity, repeatable method which allows to study the interface histology *in vivo*, and hence is suitable to study the interface healing process.

Previous studies have evaluated the interface on a smaller sample of patients, or at lower resolution with white-light confocal microscopy. Our study shows a correlation between visual acuity and interface grading. Interestingly, the deep part of the stroma, where DSAEK surgery takes place, is interested by a number of pathological changes. For example, it has been shown that fluid entering the cornea causes more swelling in the posterior than in the anterior lamellae. Also, posterior lamellae can reach higher degree of final hydration, and collagen-free regions (known as “lakes”) exist in Fuch’s dystrophy corneas which are thought to be caused by dead cells and fibril disordering. The selective distribution of fluid into the corneal stroma might reflect a different glycosaminglycan concentration in the posterior part of the cornea [13]. All these observations contribute to explain the formation of an interface haze following DSAEK, and corroborate a correlation between haze extent and visual acuity loss. To our knowledge, this is the first report finding a correlation between a published confocal HRT2 measure and BSCVA. This finding is in contrast with what previously reported by Espana et al. [17], using a different microscope (ConfoScan as opposed to HRT). However, caution should be used in comparing these two studies, as the axial resolution provided by HRT is more than double than the ConfoScan, and this could explain the different findings.

Similarly to others [17,18], we found no correlation between particle number and visual acuity. Small particles located at the interface are a common finding following DSAEK, and are also observed after LASIK surgery [19]. The origin of interface particles has been object of research: they are generally thought to be associated with the use of the microkeratome [20-22], but they were also observed after femtosecond laser application [23]. Although previous studies suggested a deleterious effect of particles on visual acuity [5], it is now becoming clear that this may not be the case [17,18]. Our study confirms that small debris should not represent a concern to the surgeon. Interestingly, we did not find any correlation between the particle number and time from surgery (Spearman correlation coefficient 0.53, even though the P value (0.06) was not extremely low). In contrast with this, Kobayashi et al. reported a progressive reduction of particle number with time [11,24]. Our findings could also be due to a difference in the materials used and/or environment encountered during the surgery and/or the relatively small size of our sample. Similarly to other authors, we did not find activated keratocytes or dendritic cells at the interface [11,17,24]. This could be due to either the low density of these cells in the posterior stroma, or to the time passed from surgery, which would allow keratocytes to become quiescent.

A correlation between interface grading and time form surgery- suggested by this study- was confirmed by other papers [11,24] implying that a healing process occurring at the interface - an actual surgical wound in the cornea - is associated with a progressive gain in visual acuity.

We would like to point out that this study also had limitations. One of these is represented by the small sample number; hence bigger samples would be needed to confirm our findings. Secondarily, other potentially useful outcomes such as topography and pachymetry were not considered. We anticipate this may be the object of future studies.

## Conclusions

In the present study, we quantified the interface quality following DSAEK surgery. We found that a good quality interface is related to a better visual acuity, and improves with time from surgery. Also, the presence and number of particles imaged at the interface is not related with visual outcome. We propose that *in vivo* LCM could be a helpful tool in the follow-up of DSAEK patients, as the study of the interface gives information potentially relevant for major clinical outcomes (such as BSCVA) together with other relevant information, such as endothelial cell count and keratocyte activation status.

## Informed consent

This study was performed with informed consent and in accordance with institutional guidelines.

## Competing interests

No financial and non-financials interests to disclose.

## Authors’ contributions

GF imaged the patients participated in image grading and drafted the manuscript. VR participated in patient recruitment. LL participated in patient recruitment and patient management. ED participated in patient imaging. CM is the senior author and this study was started and completed under his direct supervision, he coordinated the study design and helped to draft the manuscript. All authors read and approved the final manuscript.

## Source(s) of funding

Giulio Ferrari received a research grant from the Bietti Eye Foundation, IRCCS Rome, Italy. Claudio Macaluso receieved a grant from Programma di Ricerca Regione Università (DGR22422007).

Other authors have no sources of funding to declare.

## Pre-publication history

The pre-publication history for this paper can be accessed here:

http://www.biomedcentral.com/1471-2415/12/32/prepub
